# Developing the health workforce for universal health coverage

**DOI:** 10.2471/BLT.19.234138

**Published:** 2019-12-04

**Authors:** Giorgio Cometto, James Buchan, Gilles Dussault

**Affiliations:** a Health Workforce Department, World Health Organization, avenue Appia 20, 1211 Geneva 27, Switzerland.; b Instituto de Higiene e Medicina Tropical, Universidade Nova de Lisboa, Lisboa, Portugal.

## Abstract

Optimizing the management of the health workforce is necessary for the progressive realization of universal health coverage. Here we discuss the six main action fields in health workforce management as identified by the Human Resources for Health Action Framework: leadership; finance; policy; education; partnership; and human resources management systems. We also identify and describe examples of effective practices in the development of the health workforce, highlighting the breadth of issues that policy-makers and planners should consider. Achieving success in these action fields is not possible by pursuing them in isolation. Rather, they are interlinked functions that depend on a strong capacity for effective stewardship of health workforce policy. This stewardship capacity can be best understood as a pyramid of tools and factors that encompass the individual, organizational, institutional and health system levels, with each level depending on capacity at the level below and enabling actions at the level above. We focus on action fields covered by the organizational or system-wide levels that relate to health workforce development. We consider that an analysis of the policy and governance environment and of mechanisms for health workforce policy development and implementation is required, and should guide the identification of the most relevant and appropriate levels and interventions to strengthen the capacity of health workforce stewardship and leadership. Although these action fields are relevant in all countries, there are no best practices that can simply be replicated across countries and each country must design its own responses to the challenges raised by these fields.

## Résumé

Il est nécessaire d'optimiser la gestion du personnel de santé pour parvenir progressivement à la couverture sanitaire universelle. Dans cet article, nous nous intéressons aux six grands domaines d'action en matière de gestion du personnel de santé qui sont définis dans le Cadre d'action concernant les ressources humaines pour la santé: leadership; finances; politiques; éducation; partenariats; et systèmes de gestion des ressources humaines. Nous décrivons également des exemples de pratiques efficaces pour renforcer le personnel de santé, en mettant en avant l'étendue des questions que les responsables politiques et les planificateurs devraient prendre en compte. Il n'est pas possible de réussir dans ces domaines d'action en les abordant de manière séparée. Ce sont des fonctions étroitement liées qui dépendent d'une forte capacité à gérer efficacement les politiques relatives au personnel de santé. Cette capacité de gestion peut être mieux comprise sous la forme d'une pyramide d'outils et de facteurs englobant les niveaux des individus, des organisations, des institutions et des systèmes de santé, dans laquelle chaque niveau dépend de la capacité du niveau inférieur et permet d'agir au niveau supérieur. Nous nous intéressons ici aux domaines d'action qui correspondent aux niveaux des organisations ou des systèmes et qui concernent le renforcement du personnel de santé. Selon nous, il est indispensable d'analyser le cadre stratégique et les structures de gouvernance, ainsi que les mécanismes d'élaboration et de mise en œuvre des politiques relatives au personnel de santé. Cette analyse devrait permettre de déterminer les niveaux et les interventions les plus appropriés pour renforcer la capacité de gestion et de direction du personnel de santé. Bien que ces domaines d'action concernent tous les pays, aucune meilleure pratique ne peut être simplement reproduite dans tous les pays. Chaque pays doit trouver ses propres réponses aux questions soulevées par ces domaines.

## Resumen

La optimización de la gestión de la fuerza laboral sanitaria es necesaria para la realización progresiva de la cobertura sanitaria universal. La optimización de la gestión de la fuerza laboral sanitaria es necesaria para la realización progresiva de la cobertura sanitaria universal. En este documento se examinan los seis campos de acción principales de la gestión de la fuerza laboral sanitaria identificados en el Marco de Acción de Recursos Humanos para la Salud: liderazgo, finanzas, políticas, educación, asociaciones y sistemas de gestión de los recursos humanos. También se identifican y describen ejemplos de prácticas efectivas en el desarrollo de la fuerza laboral sanitaria, destacando la amplitud de los temas que los responsables de formular políticas y los planificadores deben considerar. No es posible alcanzar el éxito en estos campos de acción si se persiguen de forma aislada. Más bien, se trata de funciones interrelacionadas que dependen de una fuerte capacidad de gestión eficaz de la política de la fuerza laboral sanitaria. Esta capacidad de gestión puede entenderse mejor como una pirámide de herramientas y factores que abarcan los niveles individual, organizativo, institucional y del sistema de salud, en la que cada nivel depende de la capacidad en el nivel inferior y de las medidas de habilitación en el nivel superior. Se hace énfasis en los campos de acción cubiertos por los niveles de la organización o de todo el sistema que se relacionan con el desarrollo de la fuerza laboral sanitaria. En este contexto, es necesario realizar un análisis del entorno normativo y de gobernanza y de los mecanismos para el desarrollo y la implementación de las políticas de la fuerza laboral sanitaria, y debe guiar la identificación de los niveles e intervenciones más pertinentes y apropiados para fortalecer la capacidad de gestión y liderazgo de la fuerza laboral sanitaria. Aunque estos campos de acción son relevantes en todos los países, no hay mejores prácticas que puedan ser simplemente replicadas a través de los países y cada país debe diseñar sus propias respuestas a los desafíos planteados por estos campos.

## ملخص

يعد تحسين إدارة القوى العاملة في القطاع الصحي أمراً ضرورياً لتنفيذ التغطية الصحية الشاملة بشكل تدريجي. سوف نناقش هنا مجالات العمل الستة الرئيسية في إدارة القوى العاملة في القطاع الصحي وفقاً للتوضيح الوارد في "الموارد البشرية لإطار العمل الصحي": القيادة؛ والشؤون المالية؛ والسياسات؛ والتعليم؛ والشراكة؛ ونظم إدارة الموارد البشرية. كما نقوم كذلك بتوضيح ووصف أمثلة للممارسات الفعالة في تطوير القوى العاملة في القطاع الصحي، مع التركيز على مجموعة القضايا التي يجب أن يضعها واضعو ومخططو السياسات في الاعتبار. من غير الممكن تحقيق النجاح في مجالات العمل هذه من خلال السعي لتحقيقها بمعزل عن غيرها. بل هي وظائف مترابطة تعتمد على قدرة قوية على الإشراف الفعال لسياسة القوى العاملة في القطاع الصحي. يمكن الوصول لأفضل فهم لقدرة الإشراف تلك على أنها هرم من الأدوات والعوامل التي تشمل مستويات النظام الفردية والتنظيمية والمؤسسية والصحية، حيث يعتمد كل مستوى على قدرة المستوى أدناه، ويقوم بتمكين الإجراءات على المستوى أعلاه. نحن نركز على مجالات العمل التي تغطيها المستويات التنظيمية، أو على مستوى النظام، والتي تتعلق بتطوير القوى العاملة بالقطاع الصحي. نحن نعتبر أنه من المطلوب القيام بتحليل وتنفيذ السياسات وبيئة الحكم وآليات تطوير سياسة القوى العاملة بالقطاع الصحي، كما يجب أن نقوم بالتوجيه في تحديد المستويات الملائمة والأكثر صلة، والتدخلات المطلوبة لدعم الإشراف على القوى العاملة بالقطاع الصحي وقيادتها. على الرغم من أن مجالات العمل تلك مناسبة لكل البلدان، إلا أنه ليست هناك ممارسات مُثلى يمكن ببساطة تكرارها عبر البلدان، ويجب على كل بلد تصميم الاستجابات الخاصة بها للتحديات التي تطرحها هذه المجالات.

## 摘要

优化卫生人力管理是逐步实现全民健康覆盖的必然要求。这里我们讨论的是《卫生人力资源行动框架》中确定的卫生人力资源管理的六大行动领域：领导力；财政；政策；教育；伙伴关系；和人力资源管理系统。我们还确立并描述了发展卫生人力中有效做法的例子，强调政策制定者和规划者应注重思考问题时的广度。在这些行动领域取得成功是不可能分开进行的。相反，环环相扣、相互关联才能发挥出它们的价值，而这取决于有效管理卫生人力政策的强大能力。这种管理能力的最佳理解是由工具和因素组成的金字塔，它包括个人、组织、机构和卫生系统，每一级都取决于下一级的能力，并扶持上一级的行动。我们重点关注与卫生人力资源发展相关的组织或系统等级所涵盖的行动领域。我们认为，需要对政策和治理环境以及制定和执行卫生人力政策的机制进行分析，并应指导确定最相关和最适当的等级和干预措施，以加强卫生人力管理的管理和领导能力。尽管这些行动领域在所有国家都具有相关性，但没有一个可以适用于各个国家的最佳做法，每个国家都必须设计自己的应对措施，克服来自这些领域的挑战。

## Резюме

Оптимизация управления кадровыми ресурсами в сфере здравоохранения необходима для последовательной реализации программы всеобщего охвата услугами здравоохранения. Авторы обсуждают шесть основных областей деятельности в сфере управления трудовыми ресурсами здравоохранения, которые определены в Рамочной программе действий в области кадровых ресурсов здравоохранения: лидерство, финансирование, политику, образование, партнерство и системы управления кадровыми ресурсами. Авторы также выявляют и описывают примеры эффективных методов по развитию кадровых ресурсов здравоохранения, подчеркивая широкий спектр вопросов, которые следует учитывать лицам, формирующим политику, и специалистам по планированию. Добиться успеха в данных областях деятельности невозможно, если работать над ними изолированно. Напротив, они являются взаимосвязанными функциями, которые зависят от того, существует ли значительный потенциал эффективного руководства политикой кадровых ресурсов здравоохранения. Такой руководящий потенциал легче всего представить как пирамиду инструментов и факторов, охватывающих индивидуальный, организационный, ведомственный уровни и уровень системы здравоохранения, причем каждый уровень зависит от потенциала нижестоящего уровня и стимулирующих мер на вышестоящем уровне. Авторы уделяют особое внимание областям деятельности на организационном и общесистемном уровнях, которые связаны с развитием кадровых ресурсов здравоохранения. Они считают, что необходим анализ политики и культуры управления, а также механизмов разработки и реализации политики в области кадровых ресурсов здравоохранения, который должен послужить основанием для определения наиболее актуальных и подходящих уровней и мероприятий для укрепления потенциала управления кадрами здравоохранения и их лидерства. Несмотря на то что данные области деятельности актуальны для всех стран, универсальных методов, которые можно применять в разных странах, не существует. Следовательно, каждая страна должна разработать свои собственные решения для проблем, возникающих в указанных областях.

## Introduction

There is growing recognition that the progressive realization of universal health coverage (UHC) is dependent on a sufficient, equitably distributed and well performing health workforce.^1^ Optimizing the management of the health workforce has the potential to improve health outcomes, enhance global health security and contribute to economic growth through the creation of qualified employment opportunities.^2^

The effective management of the health workforce includes the planning and regulation of the stock of health workers, as well as education, recruitment, employment, performance optimization and retention. Health workforce management is a difficult task for many reasons. For example, there can be skills shortages and funding constraints. Health workers can also form groups (associations, unions and councils) with political and social power; such groups can defend and promote objectives and interests that are not always aligned with national health priorities and objectives. Historically, the health labour market has been highly regulated through barriers at entry and restrictions on tasks that specific health workers can perform; the most highly qualified workers have also secured significant autonomy in performing their work. The health workforce development function is part of, and therefore needs to be integrated with, health system governance and management, health sector policy and legislation, and service delivery strategies and mechanisms.

Here, we discuss the six main action fields in health workforce management identified by the Human Resources for Health Action Framework:^3^ leadership; finance, policy; education; partnership; and human resources management systems. We have adopted this framework because it is explicitly focused on actions required by policy-makers and planners (all six action fields) and managers (included in the last three action fields), as opposed to other frameworks that are based on the perspective of the individual health worker or more focused on the labour and finance elements. These six action fields are relevant in countries at all levels of socioeconomic development, including those affected by conflict and chronic complex emergencies. As a result of their intrinsic complexity, and the need to adapt interventions to the specific context and vested interests of a country, these action fields require long-term strategic vision and commitment.

**Fig. 1:**
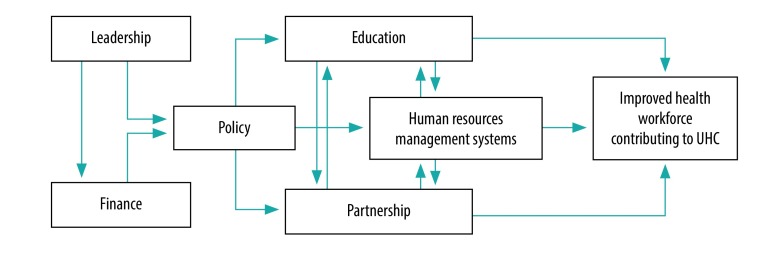
Linking of the action fields of the Human Resources for Health Action Framework to improve heath workforce management

We elucidate the logical hierarchy and links between the six different action fields (Fig. 1). We identify and describe illustrative examples of effective practices in health workforce development according to these six action fields, highlighting the breadth of issues that policy-makers and planners should consider.

UHC: universal health coverage.

Note: Adapted from *Human resources for health action framework*.^3^

## Leadership

The effective planning, development, regulation, oversight and management of the health workforce requires more than having a human resources department in a health ministry performing the bureaucratic work of processing recruitment, transfers and retirement. Effective leadership means: identifying needs, priorities and objectives; designing and implementing fitting policies; and managing interactions with other government sectors and regulatory agencies that make decisions impacting on the health workforce. The Islamic Republic of Iran, which has a Ministry of Health and Medical Education, is a rare example of a country that has formalized the coordination between the two sectors.^4^ Other government ministries will also be influential in contexts where health services are primarily delivered in the public sector; the ministries of finance and public services can impose constraints on remuneration and working conditions.

## Finance

### Mobilizing commitment and support

A critical part of the management of the health workforce is to mobilize political leadership and financial support (to ensure that policies survive leadership changes in government) and build support from stakeholder organizations. Political leadership is required for a whole-of-government approach instrumental to: (i) advocate the business case for strengthening the health workforce and mobilizing stakeholder support; (ii) marshal financial and policy support from ministries of finance, education, labour, civil service commissions, local government and the private sector; (iii) accelerate the adoption of relevant innovations; (iv) mobilize adequate financial resources to meet needs (primarily from domestic resources but, in the case of aid-dependent countries, also from development partners);^5^ and (v) overcome rigidities in public sector regulations.

Targeted funding can support the effective development of human resources for health, but overlapping sources of funds creates the risk of undermining effective coordination.^6^ The evidence suggests that sustained leadership in pursuing policy reforms and in coordinating the targeting of funds is more important than the production of planning and strategic documents; for example, over several political cycles the governments in Brazil^7^ and Thailand^8^ have been relatively successful in maintaining basic policy objectives, such as strengthening primary care, by creating sustained collaboration between ministries, national agencies, and state and local authorities.

## Policy

### Workforce planning for UHC

The planning of the health workforce should address requirements holistically, rather than by occupational groups, and be informed by population and health system current and expected future needs. Such planning should cover education policies, financing requirements, governance and management, and be a continuous process with regular monitoring and adjustment of priorities. Determining today the number and type of health workers that will be needed in 10–20 years is a complex and often inexact exercise; such a process requires both a valid picture of the current situation and a clear vision of the services that will be needed in the future. A good understanding of the dynamics of the health labour market is also a prerequisite; knowing how the participation rates, mobility patterns, aspirations and behaviour of workers will evolve is therefore critical. Certain countries have used this type of information to forecast requirements for skills and competencies in the health and social care workforce (United Kingdom of Great Britain and Northern Ireland),^9^ improve the distribution of pharmacists (Lebanon)^10^ and assess the planning implications of demographic change in the nursing workforce (Ghana).^11^

Countries with a small population face additional challenges as they cannot expect to achieve economies of scale and be totally self-sufficient, for instance in the education of highly specialized health workers. Educational functions and facilities can be pooled through intercountry collaboration in the form of bilateral or multilateral agreements. Examples of pooling resources include between-country agreements in Europe on sharing the training and development of specialist staff,^12^ and a medical school in Fiji being accessible on a cooperative basis to nationals from other Pacific island countries as a primary resource for the training of doctors.^13^

### Effective information systems

A recurrent recommendation is to build or strengthen human resources databases that provide policy-makers, planners, researchers and other potential users with valid, reliable, up-to-date and easily accessible data on the health workforce. Major international databases from the World Bank, World Health Organization (WHO) and Organisation for Economic Co-operation and Development (OECD) use data provided by their Member States, but data are provided with varying degrees of quality and completeness between countries. In most countries, there are different sources of potentially useful data (e.g. government ministries, professional councils and training institutes); setting a common definition of required data and coordinating data collection is challenging but important.^14^

With a view to standardizing data collection by countries, the WHO Regional Office for Europe, Eurostat and the OECD have combined forces to develop a joint questionnaire^15^ that includes sections on health employment and education, and health workforce migration. In addition, WHO provides guidance on a minimum data set for health workforce registry^16^ and on the development of National Health Workforce Accounts^17^ to improve data availability. Another example is the successful establishment of human resources observatories for health, as in Sudan.^18^ Independent organizations that produce research evidence to inform the health workforce policy process operate in Canada (WHO Collaborating Centre on Health Workforce Planning and Research, based at Dalhousie University in Halifax, Nova Scotia), England (Health Education England) and the United States of America (Healthforce Center at University of California, San Francisco).

## Education

### Appropriate candidates for health professional education

Health workers must have the profile, skills and behaviour that creates trust in the population and promotes demand for quality services. These criteria imply that candidates for training as health professionals must have specific characteristics, such as the ability to communicate, show empathy, be sensitive to cultural differences and work as a team. In most countries the selection of students is by academic grades; although this is a good predictor of future academic performance, it does not reveal anything about future professional performance. The University Clinical Aptitude Test is used in the United Kingdom for the selection of medical and dental applicants to assess mental attitude and behavioural characteristics consistent with the demands of clinical practice.^19^ In South Africa, there have been successful examples of more inclusive admission policies coupled with bridging programmes that support students from underprivileged backgrounds.^20^

### Competency-based education

For a decade, calls have been made to transform education programmes and learning strategies to ensure that future health workers have the required competencies for the changing burden of diseases and technological environment.^21^ Desirable competencies must be identified and aligned with population health priorities and any identified skills gaps. In many countries, this means a shift in focus towards education and training that prepares the workforce to deliver effective primary care and meet the increasing challenge of noncommunicable diseases.^22^^,^^23^ There are good examples of education for primary care practice in medicine and nursing in Portugal,^24^ South Africa^25^ and Thailand.^26^

### Adequate mix of skills

The training and deployment of a sufficient stock of health workers that comprise an optimal mix of skills may entail scaling-up the capacity and staffing of training institutions, and investing in infrastructure. Although there are generally sufficient applicants for medical studies, applicants for training in other health professions, such as nursing are sometimes insufficient. Austria, Belgium, Denmark, Germany and the Netherlands are some of the countries that have launched targeted campaigns to attract students to nursing and to other occupations with unmet needs, for example, in the fields of radiography and medical laboratory technology.^27^ An additional challenge is to attract students to less popular (but no less important) medical specialization fields such as family practice, mental health, emergency care or geriatrics. One solution to understaffing in less-popular specialties and geographical areas is to consider alternate providers. In some contexts, community-based and mid-level health workers, adequately supported by the health system, have been effective in expanding coverage and improving health service equity (e.g. in rural areas or for low-income or vulnerable groups).^28^^,^^29^

### Regulating education and practice 

The development and activation of a regulatory framework that upholds accepted standards of education and practice can include the accreditation of training programmes and institutions, the licensing and certification of health facilities and of individual health workers, and laws defining the scope of practice for each level of worker. Such a framework can also cover the regulation of work in the private sector, including dual practice (where professionals employed in the public sector can undertake work in the private sector)^30^ and the regulation of private sector education institutions, mechanisms of surveillance of practice by professional councils, and the exercise of discipline in cases of malpractice or unethical behaviour.

In many countries, some of these functions are the responsibility of independent nongovernmental organizations, such as accreditation bodies and professional councils. There is wide variation in educational requirements, regulation and scope of practice between countries and for different professions.^31^ These organizations require continuous funding to function effectively, which explains why they tend to be more developed in high-income countries and in countries with larger professional memberships.^32^ Right-touch regulation in England entails regulating only what is necessary, monitoring results, checking for unintended consequences and ensuring adherence to explicit policy objective.^33^

## Partnership

### Effective labour relations

Studies on the adverse effects of poor labour relations (e.g. between management and unions), evidenced by striking health workers, are more abundant than those on good practices in labour relations to limit such disruptions. To identify good practices, studying the experience of countries where conflict management is effective in preventing service disruption is needed, as well as identifying contributing factors to prevention. Context-specific policy recommendations have been developed to guide the management of labour relations in countries that have recently witnessed large-scale industrial action by health workers (e.g. Kenya), emphasizing the importance of mechanisms of dispute resolution.^34^ Experience from South Africa highlights the need to preserve access by the population to essential services during episodes of industrial action.^35^

## Human resources management systems

### Supporting and retaining workers

Decent work can contribute to making health systems effective and resilient, and to achieving equal access to quality health care.^36^ Decent work may have different meanings depending on the context; a good indicator of its existence is the capacity of provider organizations to recruit the staff they need and to retain them. In the USA, so-called magnet hospitals report higher nurse satisfaction, less staff turnover, higher patient satisfaction and better health outcomes.^37^^,^^38^ In Portugal, family health units are self-constituted teams of physicians, nurses and administrative secretaries, which demonstrate better worker and user satisfaction, coverage and health outcomes than traditional health centres that are staffed through public recruitment and where professionals operate in a more rigid administrative environment.^36^ What these examples from the USA and Portugal have in common is that workers have more autonomy, work in teams and feel respected; management is participative; and innovation is valued. Good practices, such as creating a more family-friendly environment (e.g. offering flexible hours to mothers of young children and providing access to child day-care services) or adapting working conditions for older workers to prevent early retirement, also show positive results in attracting and retaining workers in health facilities.^23^ Deliberate efforts to create a positive practice environment, with a focus on involving staff in decision-making and assessing workplace priorities, has translated into the improved motivation and performance of health workers in several low- and middle-income countries, specifically Morocco, Uganda and Zambia.^39^

### Underserved geographical areas

The attraction of health workers to rural, isolated or otherwise underserved areas, and the retention of these workers once recruited, requires a range of strategies, including: targeted education admission policies to attract candidates from underserved zones; packages of financial, professional (mentorship, networking and continuing education) and quality-of-life incentives; regulatory reforms; and bonding contracts in exchange for educational support costs.^40^ Specific policy interventions include: compulsory service in disadvantaged areas after the completion of studies, for example in South Africa;^41^ development of a role intended to provide care in rural and/or remote areas, for example in aged care nurse practitioner in Australia^42^ and surgical technicians in Zambia;^43^ an emphasis on rural experience in medical education provision; and the use of financial incentives to retain staff, for example in Cambodia, China and Viet Nam.^44^

### Managing emigration

Some high-income countries rely on active international recruitment, which can exacerbate staff shortages in lower-income source countries. Emigration flows can reach high levels from some low- and middle-income countries where working conditions are perceived as poor. These flows can be mitigated by the use of bilateral agreements, which define the conditions under which foreign workers, typically physicians and nurses, will be employed in destination countries, as well as the benefits both countries would gain from the agreement, as recommended by the WHO Global Code of Practice on the International Recruitment of Health Personnel.^45^ An example of such an agreement is that between Germany and Viet Nam, signed in 2012, in which gaps are addressed in geriatric care nurses in the destination country (Germany), and training and employment opportunities are provided for health personnel of the source country (Viet Nam).^46^ Outflows from higher-income countries can be beneficial during periods of high unemployment or underemployment, which was the case during times of austerity in Greece, Portugal and Spain.^47^

Very few studies in the literature assess good practices to prevent the emigration of health workers. The increase in remuneration of physicians in Ghana in 2008 appeared to reduce the rate of emigration by 10%, principally among physicians younger than 40 years (potential emigrants) but not of older physicians.^48^ Hungary adopted a series of measures such as pay increases and scholarships for specialty training in exchange for 10 years of work in public services, but with only limited success.^49^

## Discussion

We have discussed the six different action fields under the purview of health sector policy-makers, planners and managers, focusing on the system-wide or organizational environmental factors that relate to health workforce development. Other factors exist outside the control of policy-makers in the health sector, which in turn have a fundamental role in determining the political, technical and financial feasibility and sustainability of health workforce policies and actions. While recognizing their importance, these factors fall outside the scope of this paper.

**Fig. 2:**
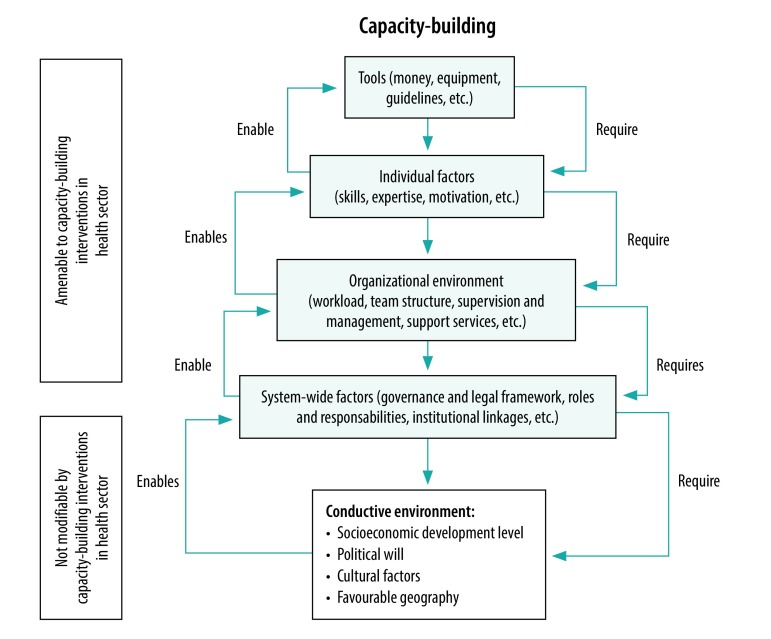
Hierarchy of needs in capacity building for effective stewardship of human resources for health

Note: Adapted from Potter C & Brough R, 2004, to illustrate relevant health workforce policy levers and external factors.^50^

Although the evidence base for the six action fields identified by the Human Resources for Health Action Framework is limited, it is still sufficient in each individual action field to warrant a dedicated review. These action fields are not strategies that can be pursued in isolation. Rather, they are interlinked functions that depend on a strong capacity for the effective stewardship of health workforce policy, as illustrated in Fig. 1. This capacity can best be understood as a pyramid of tools and factors, encompassing the individual, organizational, institutional and health system levels, where the success of each level depends on capacity at the level below and enables actions at the level above (Fig. 2).^50^

In chronic and complex emergencies and in countries emerging from conflicts that have severely limited the capacity of pre-existing governance management, priority should arguably be given to essential governance functions and to the mobilization of political commitment. Appropriate governance underpins success in other areas and is required to guarantee the functioning of the system at its most basic level, for example: in establishing (if not already in existence) a mechanism for health workforce policy dialogue and planning, a system to dynamically monitor health workforce stock and distribution, and a fund pooling mechanism for the sustainable and integrated financing of the health workforce; revamping mechanisms for the execution of agreed health workforce policies by subnational health administrations; and reinstating a functional payroll while removing the records of both ghost workers and health workers who may have been added during the period of crisis, but who are no longer part of the workforce.

An analysis of the policy and governance environment and of mechanisms for health workforce policy development and implementation is required, and should guide the identification of the most relevant and appropriate levels and interventions to strengthen the capacity of health workforce stewardship and leadership. Remembering that there are no best practices that can simply be replicated across all countries, responses to the challenges raised by these action fields are context-specific and each country must design its own.
